# The kinome of *Phytophthora infestans *reveals oomycete-specific innovations and links to other taxonomic groups

**DOI:** 10.1186/1471-2164-11-700

**Published:** 2010-12-09

**Authors:** Howard S Judelson, Audrey MV Ah-Fong

**Affiliations:** 1 Department of Plant Pathology and Microbiology, University of California, Riverside, California 92521 USA

## Abstract

**Background:**

Oomycetes are a large group of economically and ecologically important species. Its most notorious member is *Phytophthora infestans*, the cause of the devastating potato late blight disease. The life cycle of *P. infestans *involves hyphae which differentiate into spores used for dispersal and host infection. Protein phosphorylation likely plays crucial roles in these stages, and to help understand this we present here a genome-wide analysis of the protein kinases of *P. infestans *and several relatives. The study also provides new insight into kinase evolution since oomycetes are taxonomically distant from organisms with well-characterized kinomes.

**Results:**

Bioinformatic searches of the genomes of *P. infestans*, *P. ramorum*, and *P. sojae *reveal they have similar kinomes, which for *P. infestans *contains 354 eukaryotic protein kinases (ePKs) and 18 atypical kinases (aPKs), equaling 2% of total genes. After refining gene models, most were classifiable into families seen in other eukaryotes. Some ePK families are nevertheless unusual, especially the tyrosine kinase-like (TKL) group which includes large oomycete-specific subfamilies. Also identified were two tyrosine kinases, which are rare in non-metazoans. Several ePKs bear accessory domains not identified previously on kinases, such as cyclin-dependent kinases with integral cyclin domains. Most ePKs lack accessory domains, implying that many are regulated transcriptionally. This was confirmed by mRNA expression-profiling studies that showed that two-thirds vary significantly between hyphae, sporangia, and zoospores. Comparisons to neighboring taxa (apicomplexans, ciliates, diatoms) revealed both clade-specific and conserved features, and multiple connections to plant kinases were observed. The kinome of *Hyaloperonospora arabidopsidis*, an oomycete with a simpler life cycle than *P. infestans*, was found to be one-third smaller. Some differences may be attributable to gene clustering, which facilitates subfamily expansion (or loss) through unequal crossing-over.

**Conclusion:**

The large sizes of the *Phytophthora *kinomes imply that phosphorylation plays major roles in their life cycles. Their kinomes also include many novel ePKs, some specific to oomycetes or shared with neighboring groups. Little experimentation to date has addressed the biological functions of oomycete kinases, but this should be stimulated by the structural, evolutionary, and expression data presented here. This may lead to targets for disease control.

## Background

Protein kinases regulate numerous cellular processes including mitosis, communication, differentiation, metabolism, and transcription. They constitute the largest protein family in most single-celled and multicellular eukaryotes, underscoring the ubiquitousness of phosphorylation as a control mechanism. Nearly all protein kinases share a common ancestry, belonging to the eukaryotic protein kinase (ePK) superfamily [1]. These contain a core domain of about 250 amino acids which catalyzes the phosphorylation of serine, threonine, or tyrosine. Protein phosphorylation in eukaryotes is also mediated by some proteins not related closely to ePKs, including atypical protein kinases (aPKs) that act on serine or threonine and bacterial-like histidine kinases [2,3].

ePKs are typically categorized into nine families based on the sequences of their catalytic domains [1]. The AGC, CAMK, CMGC, CK, OTHER, RGC, STE, and TKL families, which are described in more detail in Results and Discussion, primarily phosphorylate serine and threonine, while the TK family acts mainly at tyrosine, although some ePKs can modify all three residues [1,4]. The further classification of ePKs into subfamilies is possible using features of the catalytic region as well as non-catalytic accessory domains [5]. The latter comprise regulatory modules, affect substrate binding, determine subcellular localization, or allow the kinases to serve as scaffolds for multipeptide complexes. The shuffling of non-catalytic domains is likely a major feature behind the diversification of eukaryotic species.

Interspecific comparisons have shown that while the major ePK families predate the eukaryotic radiation, significant changes occurred during evolution. Examples include the loss of TKL kinases from yeasts, the enlargement of receptor-like kinase subfamilies in plants, the birth and death of subfamilies in metazoan lineages, and the appearance of TKs as a mostly metazoan-specific feature [6-8]. Having data from diverse eukaryotes is important for understanding kinase evolution, and testing conclusions from early studies based on limited kinomes. For example, the greater abundance of TKs in humans than fruit flies and their absence from yeasts and slime molds once led to the proposal that TKs are linked to complex multicellular life, but then the single-celled protist *Monosiga brevicollis *was found to have more TKs than animals [9,10]. Similarly, the conclusion that most organisms devote a similar fraction of their transcriptome to ePKs, about 2%, had to be altered when this value in the ciliate *Paramecium tetraurelia *was found to be >3-fold higher [11]. Also, plant data revealed new classes of calcium-dependent kinases that do not follow the metazoan regulatory paradigm [6,12].

Stramenopiles (heterokonts) comprise a major eukaryotic kingdom that lacks close taxonomic affinity to organisms with well-characterized kinomes, and thus offer opportunities to learn more about the evolution of these proteins. Stramenopiles include many important pathogens and saprophytes in the oomycete ("water mold") group, as well as diatoms and brown algae, and are part of a larger group called Chromalveolates [13,14]. This paper focuses on the kinome of the oomycete *Phytophthora infestans*, the potato late blight agent [15]. Its life stages include vegetative hyphae, sexual structures, and asexual spores including flagellated zoospores [16]. Protein kinases have been shown to participate in the growth and differentiation of oomycetes [17]. Here we describe the *P. infestans *kinome, including its content of kinases with novel domains and oomycete-specific subfamilies, its expression pattern, and evidence of its diversification from the kinomes of related taxa.

## Results and discussion

### Discovery of P. infestans protein kinases

To identify genes encoding ePKs and aPKs the draft genome [18] was explored for sequences encoding the relevant Pfam domains and by BLAST using representative animal, fungal, and plant kinases. An iterative process of rechecking the database using diverse *P. infestans *sequences helped raise the probability of detecting kinases deviated from those of other eukaryotes, or having erroneous gene models. Particular care was used to evaluate the structure of each gene model. More plausible models were developed for about two-thirds of genes based on matches to expressed sequence tags, comparisons to sequences from *P. ramorum *and *P. sojae*, and RT-PCR in the case of genes of particular interest.

Genes encoding 354 ePKs and 18 aPKs (including 3 histidine kinases) were identified in *P. infestans*, and their features are summarized in Additional File 1 Tables S1 and S2. Not included in the above tallies or the later sections of this paper are four pseudogenes and five genes that appeared to have been artificially duplicated in the assembly. None of the pseudogenes contain A-rich sequences at their 3' termini, suggesting that they are not processed (retrotransposed) pseudogenes despite the abundance of retroelement-like sequences in the *P. infestans *genome [17].

The *P. infestans *kinome appears very similar to those of two other *Phytophthora *spp. for which draft genome sequences are available publicly, *P. ramorum *and *P. sojae *[19]. A detailed comparison was not undertaken due to the challenge of correcting gene models in the latter two species, although it appears that the number of kinases are similar. For example, using an *E *< 10^-10 ^criterion for matches against a HMM for the ePK catalytic domain (pfam00069), and after eliminating presumptive artificially duplicated genes, *P. ramorum *and *P. sojae *are estimated to contain 352 and 354 ePKs, respectively.

### Classification of P. infestans ePKs

The 354 *P. infestans *ePKs were grouped into the major families defined by Hanks and Hunter [1]. This entailed matching their catalytic domains against HMM models of each family and using BLAST to compare them against metazoan ePKs. Classifications were confirmed and ambiguities resolved by phylogenetic analyses with human, plant, and yeast ePKs. Of the eleven subdomains (I to XI) shown to represent conserved features of the kinase catalytic region [1], particular note was made of the composition of subdomains VIb and VIII, which are believed to determine substrate specificity. These generally matched the established paradigms, with some divergence especially in subdomains VIII of the CK1, CAMK, and TKL families (Additional File 2 Fig. S1).

**Figure 1:**
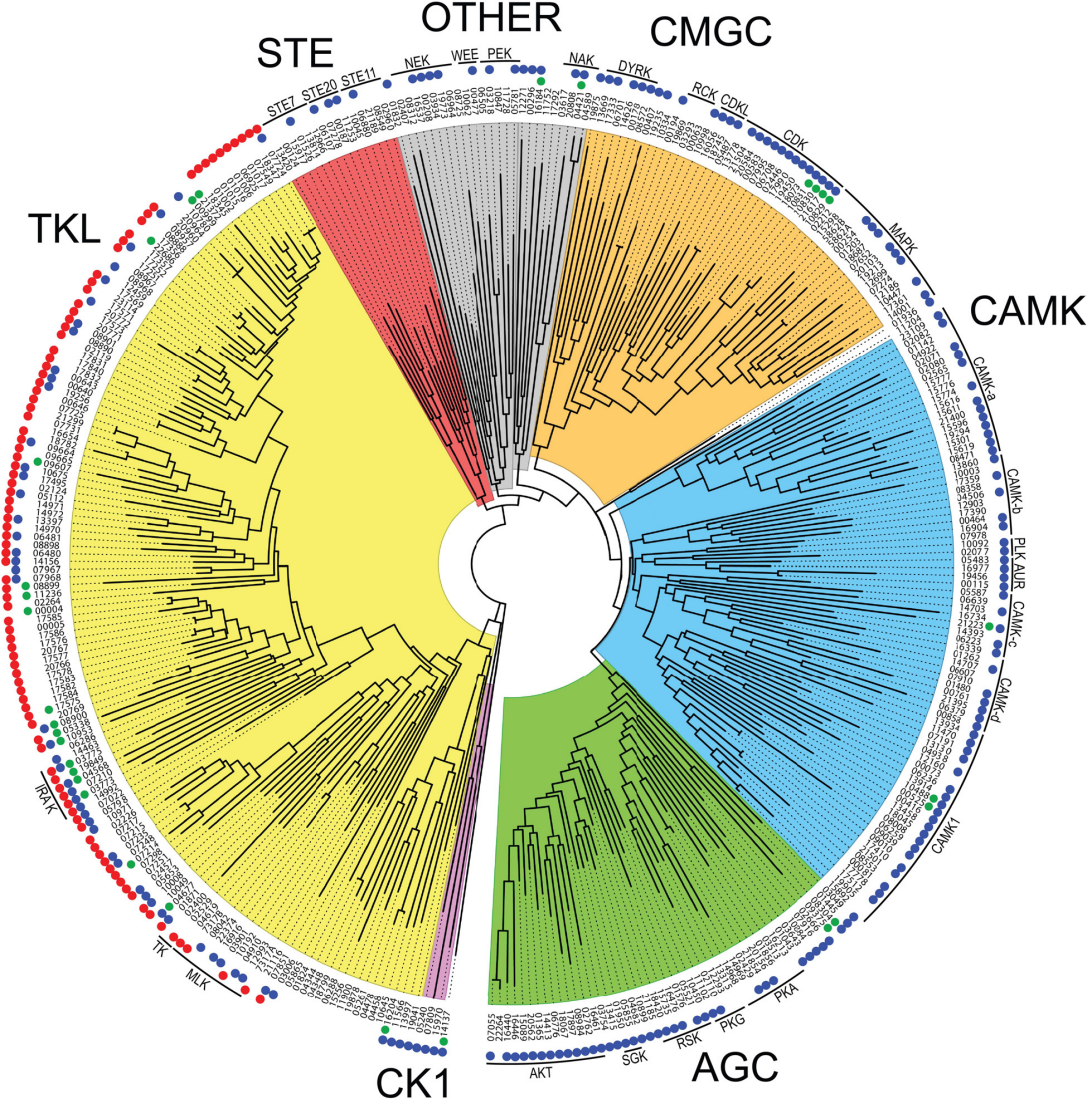
**Phylogram of *P. infestans *ePKs**. Illustrated is a maximum likelihood tree based on catalytic domains. The major groups are highlighted by different colors (AGC, CAMK, CK1 CMGC, OTHER, STE, TKL). Significant subfamilies are noted using the traditional nomenclature, except for the CAMKa-d groups which are specific to this study. Two putative TKs are included within the TKL group, and the catalytic domains of dual-kinase proteins were analyzed separately (noted by A and B suffixes on gene names). Proteins with predicted inactive catalytic regions are noted with a green dot next to the gene number. Blue dots mark proteins with clear orthologs in *H. arabidopsidis*. Red dots indicate proteins with transmembrane domains.

A phylogram of the 354 *P. infestans *ePKs that portrays their assignments to the major groups is presented Figure 1 and that information is also summarized in Table 1. The table also includes data from representative plant, animal, yeast, apicomplexan, ciliate, and slime mold kinomes along with data for two other stramenopiles, the diatom *Thalassiosira pseudonana *and the oomycete *H. arabidopsidis*, which is a downy mildew pathogen. The latter two datasets were generated for this study using the same mining scheme applied to *P. infestans*, although gene models were not corrected. It should be noted that our size estimate of the *T. pseudonana *kinome is smaller than in a previous report (152 versus 190; [20]). In total, ePKs account for 2.0% of total *P. infestans *genes, which compares to 1.3 to 6.6% for the other eukaryotes in Table 1. Details of each family in *P. infestans *are presented in later sections, except for receptor guanylyl cyclase (RGC) kinases which were not detected and are believed to be metazoan-specific [10].

**Table 1:** Content of ePKs in selected eukaryotes.

	Genes in subfamily^a^										
			
Species	AGC	CAMK	CK1	CMGC	RGC	STE	TK	TKL	OTHER	Total ePKs	% of genes^b^
*P. infestans*	47	70	3	46	0	15	2	137	34	354	2.0
*H. arabidopsidis*	36	46	3	30	0	6	0	58	27	207	1.3
*T. pseudonana*	29	52	4	25	0	5	0	14	23	152	1.3
*P. falciparum*	5	13	1	18	0	0	0	5	23	65	1.5
*P. tetraurelia*	635	970	165	322	1	118	2	21	380	2614	6.6
*O. sativa*	59	163	33	151	0	73	0	1387	31	1897	4.7
*D. discoideum*	27	21	2	28	0	44	0	68	71	261	2.1
*S. cerevisiae*	17	21	4	34	0	18	0	0	36	130	2.2
*H. sapiens*	63	74	12	63	5	47	90	43	81	478	1.4

^a^Classifications for *P. infestans, H. arabidopsidis*, and *T. pseudonana *are from this study. Assignments for the other species are from the literature [10,11,30,59].

^b^Based on estimated gene contents of 17,997 for *P. infestans *(Broad Institute v. 2); 15,551 for *H. arabidopsidis *(Virgina Bioinformatics Institute v. 8.3); 11,390 for *T. pseudonana *(Joint Genome Institute v. 3), 39,642 for *P. tetraurelia *(v. 1), 40,577 for *O. sativa *(MSU v. 6.1), 12,500 for *D. discoideum *(v. 2), 5,804 for *S. cerevisiae*; and 34,316 for H. sapiens (UCSC build 37.1).

Three ePKs (gene models PITG_04344, PITG_05862, and PITG_07317) contain two tandem ePK domains. Such composite kinases have been reported previously in animals and plants. The two ePK domains within each of the *P. infestans *proteins belong to the same family. Orthologs of these dual-kinase proteins exist in *P. ramorum *and *P. sojae*, but not *H. arabidopsidis*.

### Catalytically inactive kinases

A fraction of ePK-like proteins in most organisms are predicted to be inactive since they lack one or more of the amino acids that are required for catalytic activity. These include an aspartate in the HRD motif of subdomain VIb of the catalytic domain that is the catalytic residue, the aspartate in the DFG motif of subdomain VII that binds the Mg^2+ ^ion that coordinates ATP in the ATP-binding cleft, and a lysine in the VAIK motif of subdomain II (or in subdomain I) that also binds the ATP. Rather than being useless, these proteins are postulated to act as scaffolds for signal complexes or have other regulatory functions [21].

In *P. infestans *32 ePKs or 9% of the total appear to be incapable of phosphorylation, which is nearly the same fraction seen in organisms as diverse as humans, slime molds, and trypanosomes [10,21,22]. They include 2 AGC, 3 CAMK, 5 CMGC, 19 TKL, and 3 OTHER kinases. These are marked by green dots in Figure 1 and noted in Additional File 1 Table S1. *P. ramorum *orthologs of 25 of these are also predicted to be inactive, and this conservation suggests that most have a cellular function.

### Accessory Domains in ePKs

Sixty-eight *P. infestans *ePKs or 19% of the total contain domains other than the kinase catalytic region (Table 2; Additional File 1 Table S1). This is substantially less than in humans, where over 50% contain additional domains, many of which have regulatory functions [7]. This suggests that a greater fraction of *P. infestans *ePKs are controlled at the level of mRNA, and as described later over two-thirds are transcriptionally regulated during the life cycle. The function of many of the domains are discussed later in combination with descriptions of specific ePK families.

**Table 2:** Non-kinase domains in ePKs from P. infestans

Pfam Domain	No. of Proteins^a^	Represented Families	Function	Novel?^b^
Ankyrin	3	AGC, TKL	higher order structure	
Arb2	2	AGC	chromatin association	✓
Armadillo	1	TKL	protein interaction	
C2	1	AGC	Ca^2+^-dependent membrane targeting	
cNMP binding	8	AGC, CAMK	cyclic nucleotide binding	
cyclin N, C	4	CMGC	cell cycle control	✓
DEP	5	AGC, TKL	protein targeting and interaction	
EF hand	3	CAMK, OTHER	calcium binding	
FYVE	2	AGC	zinc binding	
HEAT	1	OTHER	protein interaction	
KA1	4	CAMK	kinase associated, unknown	
LRR	16	TKL	protein interaction	
NAF	2	CAMK	unknown	
PAS	1	CMGC	signal sensor	✓
PDZ	1	TKL	protein interaction	
PH	9	AGC, CAMK, CMGC	phosphatidylinositol binding	
PhoD	1	STE	phosphatase	
PP2C	4	AGC, CAMK, OTHER	protein phosphatase	
PX	3	AGC	phosphoinositide binding	
Response Reg	1	OTHER	two-component signaling	
RhoGAP	1	STE	GTPase activating protein	✓
RWD	2	OTHER	unknown	
RyR	1	CAMK	unknown	✓
SAM	2	STE, TKL	protein interaction	
SET	1	CMGC	protein interaction	
TPR	1	TKL	protein interaction	
UBA/TS-N	1	CAMK	ubiquitin binding	
WD40	1	OTHER	protein interaction	
WW	1	CMGC	protein interaction	✓
ZF-B	1	STE	zinc binding	

^a ^If a protein contains more than one given domain it is counted only once in that category.

^b ^Indicates combination of kinase domain and accessory domain not reported previously outside of *Phytophthora*.

**Figure 2:**
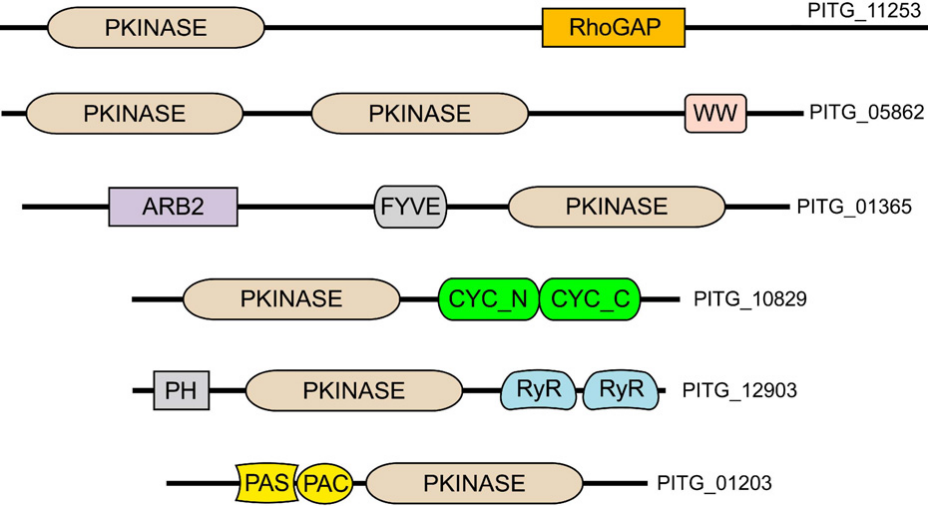
**Novel domain combinations in *P. infestans *ePKs**. Indicated are six proteins that contain accessory domains not described previously for ePKs, drawn approximately to scale. The novel domains are RhoGAP, a domain found on GTPase activating proteins; the WW protein interaction domain, which was found on a dual-kinase protein; the Arb2 domain which associates with chromatin and may influence histone methylation and siRNA formation; the N and C-terminal alpha fold domains of cyclins, cyc_N and cyc_C, which may be cell cycle control or protein interaction domains; the RyR ryanodine receptor domain, which are found on some Ca^2+ ^channels and other proteins but have unknown functions; and the PAS signal sensor domain. The latter is located just upstream of PAC, which is a structural domain that is believed to contribute to the activity of the PAS fold. PITG_12903 also contains a predicted phosphatidylinositol-binding PH (Pleckstrin Homology) domain, which is commonly found on ePKs, as is the zinc and phosphatidylinositol 3-phosphate-binding FYVE domain shown on PITG_01365.

It is notable that six domains were found that were not associated previously with ePKs in other organisms, based on searches of the Pfam database and Genbank. Examples of proteins containing an ePK domain along with novel accessory domains are shown in Figure 2. The unusual domains include the Arb2, cyclin, PAS signal sensor, and RhoGAP domains; a previous study also reported a RhoGAP-containing ePK in *P. sojae *[23]. The PAS-containing protein, PITG_01203, is particularly interesting due to its potential role in environmental sensing, which is important in the life cycle of *P. infestans*. The match to the PAS domain appears to be authentic since it is followed by a PAC domain, which contributes to PAS function. Phylogenetic analysis suggests that the PAS domain in PITG_01203 is not derived from those found in the histidine kinases of *P. infestans*, an aPK group that is described later in this paper.

Transmembrane domains were predicted for 99 of the *P. infestans *ePKs (Figure 1 red circles; Additional File 1 Table S1), ranging in number from one to nine per protein. Most of these proteins also contain recognizable signal peptide or signal anchor domains. All are in the TKL family, which as described later contains a novel family of receptor-like kinases. In addition, four proteins are predicted to contain signal peptides without transmembrane domains.

### AGC family of P. infestans

*P. infestans *encodes 47 AGC kinases, a group named after Protein Kinases A, G, and C. In other organisms, this group includes cyclic nucleotide or phospholipid-regulated kinases, G-protein coupled kinases, ribosomal protein S6 kinases, and related proteins [24]. This represents 13% of the ePK kinome, which is the same fraction as observed in humans based on the annotation of Manning et al. [7] and the number of genes in the current build of that genome, but less than in *T. pseudonana *(19%) and *H. arabidopsidis *(18%). Since *P. infestans *and *H. arabidopsidis *are both oomycetes this difference is notable, and a later section of this paper focuses on other differences between their kinomes. The composition of each ePK subfamily in these two oomycetes is also compared in Table 3. A detailed description of each AGC kinase in *P. infestans *is presented in Additional File 1 Table S1, along with the rest of the ePKs.

**Table 3:** ePK subfamilies in P. infestans and H. arabidopsidis

		Number of kinases				Number of kinases	
					
Family	Subfamily	*P. infestans*	*H. arabidopsidis*	Family	Subfamily	*P. infestans*	*H. arabidopsidis*
AGC	AKT/PKB	20	20	TK	-	2	0
	GRK	1	0				
	MAST	1	1	TKL	MLK/LRRK	28	15
	NDR	1	1		STRK/RIPK	2	2
	PKA	7	4		IRAK	5	5
	PKG	6	1		OS1	14	2
	RSK	6	3		OS2	25	10
	SGK	3	3		OS3	33	7
	unclassified	2	3		OS4	21	10
					other LRR	7	4
CAMK	CAMK1	27	19		OS4	21	10
	CAMK-a	18	14		unclassified	2	2
	CAMK-b	8	2				
	CAMK-c	9	6	OTHER	AURORA	4	3
	CAMK-d	6	6		NAK	4	1
					NEK	7	3
CMGC	CDK	17	16		PEK	5	5
	CDKL	4	0		POLO	2	2
	CK2	1	1		SCY1	1	1
	CLK	1	1		ULK	2	2
	DYRK	5	5		VSP15	1	1
	GSK	1	1		WEE	2	2
	MAPK	15	8		WNK	1	1
	RCK	3	0		unclassified	5	3
	SRPK	1	1				

STE	STE7	6	3				
	STE11	5	1				
	STE20	4	3				

Assignments are based on phylogenetic analysis of oomycete proteins against human ePKs, with a few exceptions. For CAMK, the CAMK1 group clusters well with that human subfamily, but others could not be unambigously assigned and are instead placed into four oomycete-specific clades (a to d). For the TKL family, the MLK/LRRK subfamily combines MLK and LRRK-like kinases, since these were not clearly separated in phylogenetic analyses, and the same is true for the RIPK/STRK subfamily.

The most abundant AGC subfamily within *P. infestans *is the AKT group, which has 20 members. In other organisms these are commonly regulated by phosphoinositides, but only six of the *P. infestans *proteins contain recognizable N-terminal PH or PX domains that typically bind such compounds. Next in abundance were members of the cGMP and cAMP-regulated PKG and PKA subfamilies, and the ribosomal protein S6 kinase (RSK) subfamily. Although each *Phytophthora *spp. contains 24 or more G-protein coupled receptors (GPCR), only one GPCR-regulated kinase (GRK) was detected, PITG_16476. This low number of GRKs might be explained by the observation that some *P. infestans *GPCRs contain a phosphatidylinositol phosphate kinase domain which may be capable of protein phosphorylation [25]. Members of the MAST, NDR, RSK and SGK subfamilies were also detected. While three AGC kinases had catalytic domains that appeared nearly equidistant between PKA and PKC (PITG_03513, PITG_16213, PITG_16461), none bore the C1 or C2 ligand-binding domains typical of PKC in other taxa. PKC is also absent from *Dictyostelium discoideum *and plants but in metazoans and yeast, which is consistent with the suggestion that it arose late in evolution [10].

The detection of cGMP-regulated ePKs in *P. infestans *is of particular interest. Their absence from plants, *D. discoideum*, and yeast led to a prior suggestion that PKG is metazoan-specific [10]. Since our identification of the PKG proteins was based on the traditional approach of studying the ePK catalytic domain where differences between different AGC subfamilies are subtle, the possibility of misclassification was considered. However, all six predicted PKGs contain the expected cyclic nucleotide binding domains. We can also detect PKG in the sequenced genomes of ciliates such *Paramecium tetraurelia*, apicomplexans such as *Toxoplasma gondii*, and diatoms such as *T. pseudonana*. The shared occurrence of PKG in these species is not surprising since these groups and stramenopiles reside on the same branch of the eukaryotic tree, with the ciliates and apicomplexans closer to the root and the diatoms closer to the tip [14,26]. Due to this taxonomic affinity, comparisons between oomycete, apicomplexan, and ciliate kinomes will be highlighted in many of the following sections of this paper.

Two of the predicted PKG proteins, PITG_08304 and PITG_09375, did not cluster near the rest in Figure 1. Both contain inactive kinase domains, as well as protein phosphatase and cNMP-binding domains. Predicted proteins containing this curious combination of domains, in the same orientation and including the inactive kinase, can also be found in plants but not apicomplexans or ciliates. While their functions are uncharacterized, it is possible that the kinase-like domain helps bind substrates of the phosphatase. This connection between *P. infestans *and plant ePKs is notable. While stramenopiles do not have overall taxonomic affinity with plants, they are thought to include genes transferred from a photosynthetic red algal endosymbiont which could be shared with the plant lineage [14,19].

One AGC kinase, PITG_06776, is also unusual in that it contains a predicted N-terminal Arb2 domain. No other known ePK bears this domain, which is seen in proteins that bind chromatin and are involved in siRNA generation. The domain is also found in orthologs in *P. ramorum*, *P. sojae*, and *H. arabidopsidis *but an ortholog is not present in apicomplexans, ciliates, or diatoms.

### CAMK family

Seventy *P. infestans *ePKs are within this category, making it the second largest (Additional File 1 Table S1). The family was defined initially by mammalian kinases that contain a catalytic domain that is activated when the Ca^2+^-binding EF-hand protein calmodulin binds to a downstream association domain. While the family's name is thus an abbreviation for Ca^2+^/calmodulin-regulated kinases, not all members of the family in eukaryotes have this traditional structure or function. For example, plants, ciliates, and apicomplexans instead contain proteins that seem to have evolved from a fusion between CAMK and calmodulin genes, leading to a gene product with both kinase and EF-hand domains [12,27]. Widely distributed in eukaryotes is another CAMK subfamily that lacks both calmodulin-binding or calmodulin-like domains, which is named after the sucrose non-fermenting SNF1 kinase of yeast. Due to the diversity within the CAMK group, some researchers refer to it as the calcium-dependent protein kinase (CDPK) or the CDPK-SNF1 related kinase family (CDPK-SnRK).

The relative size of the CAMK family in *P. infestans *(19% of ePKs) slightly exceeds that of metazoans, where Ca^2+ ^is known to play major roles in cellular regulation. However, it is also demonstrated that Ca^2+ ^controls many stages of oomycete development [17,28]. Nevertheless, less than 10% of *P. infestans *ePKs in the CAMK family bear accessory domains consistent with regulation by Ca^2+^. About half contain sizeable regions C-terminal to the catalytic site, with a mean size of 110 aa, but none resemble the calmodulin-association regions of mammalian CAMKs.

Four *P. infestans *proteins contain kinase and C-terminal EF-hand domains in a single peptide, namely PITG_00525, PITG_08008, PITG_12271, and PITG_13934. Such proteins thus resemble the CDPK subfamily found previously only in plants, ciliates, and apicomplexans [29-31]. This supports the concept of genes being passed to this eukaryote lineage from a red algal symbiont. The presence of only four CDPKs in *P. infestans *is surprisingly small. By comparison, the plant *Arabidopsis thaliana*, the apicomplexan *T. gondii*, and the ciliate *P. tetraurelia *contain about 42, 16, and 101 members of this class, respectively. The possibility was considered that additional kinases containing EF-hands were not detected due to the evolutionary distance between *P. infestans *and organisms used previously to define the EF-hand domain. To test this, a comparison was made of position-specific sequence matrices (PSSMs) from 100 EF-hands from 42 *P. infestans *proteins and those in the SMART database. The two PSSMs were very similar, suggesting that most EF-hands were detected and *P. infestans *only has four ePKs in this class. Three of these contain four EF-hand domains. In the diatom *T. pseudonana *we detect only one CDPK-like kinase, which contains two EF-hands. Visinin-like CDPK kinases, which bear three EF-hands and are found in plants, were not detected within *P. infestans *or the diatom.

While classic CDPKs contain EF-hand domains C-terminal to the catalytic domain, PITG_08008 contains N-terminal EF-hand domains which has not been described for any ePK. A PH domain also resides between the EF and catalytic domains, and the novel structure of this gene was confirmed by RT-PCR. PITG_08008 does not cluster with the plant-like CDPKs of *P. infestans *(Figure 1). Its catalytic domain is instead closest phylogenetically to PITG_06259, which also has a PH domain. This suggests that PITG_08008 arose from a PITG_06259-like ancestor that fused with EF-hand domains. It should be noted that the EF domains are a weak match to the eukaryotic consensus, and when we performed binding assays between recombinant PITG_08008 and ^45^Ca^2+ ^negative results were obtained.

Two *P. infestans *kinases, PITG_01480 and PITG_21395, resemble the SnRK3 subclass of plant SNF1-related kinases that are regulated by Ca^2+ ^through calcineurin B-like (CBL) sensors. CBLs have been described as a plant-specific lineage of Ca^2+ ^binding proteins that resemble the regulatory B-subunit of calcineurin and the neuronal Ca^2+ ^sensor of animals, and SnRK3 has also been described as being plant-specific [12]. *P. infestans *nevertheless contains a CBL protein, PITG_02011, in addition to the two SnRK3-like kinases. The latter contain a N-terminal catalytic domain followed by a CBL-binding NAF domain, and a C-terminal KA1 kinase associated domain. While one *P. infestans *protein (PITG_21395) has a good match to the NAF domain signature, the other (PITG_01480) is more diverged and might be nonfunctional. Unlike the CDPK subfamily described in the preceding paragraph that is also in ciliates and apicomplexans, SnRK3 appears to be plant and oomycete-specific, with no members in *T. gondii *or *P. tetraurelia*. No SnRK3-like protein was detected in the databases of the diatoms *T. pseudonana *or *Phaeodactylum tricornutum*.

Plants contain two other SnRK subfamilies which are not regulated by Ca^2+ ^[12,27]. Instead, SnRK1 and SnRK2 are controlled by proteins interacting with C-terminal ubiquitin association (UBA/TS-N) or acidic domains, respectively. These proteins also contain a KA1 domain, like SnRK3. *P. infestans *lacks an obvious SnRK2 but encodes an SnRK1 based on the presence of UBA/TS-N and KA1 in PITG_14707. PITG_00858 is related but lacks obvious UBA/TS-N, NAF, or acidic domains.

A curious connection to Ca^2+ ^signaling is observed in PITG_12903, which contains two C-terminal RyR (ryanodine receptor) domains. These motifs have not been reported previously in any ePK. Their function is unknown but interestingly are a feature of many Ca^2+^-activated ion channels [32]. Orthologs can also be detected within *P. ramorum *and *P. sojae*, but not *H. arabidopsidis*. This protein therefore appears to be an innovation in *Phytophthora*, although alternatively it could have been lost from the downy mildew. In cases such as this when *H. arabidopsidis *is reported to lack an ortholog of a *P. infestans *gene, this conclusion is based on searches of gene models, predicted noncoding regions of assemblies, and unpaired reads since not all gene models or assemblies may be correct.

Twenty-one CAMK kinases contain very short domains C-terminal to the catalytic region (<10 aa) that are unlikely to bind another protein. Six of these also contain very short regions upstream of the catalytic domain, with a typical example being PITG_15777 which extends 3 and 6 amino acids up- and downstream of the catalytic domain. While this organization superficially resembles the plant PEP carboxylase kinase subfamily (PPCK; [27]), such *P. infestans *proteins do not cluster with PPCKs in phylogenetic analysis. Nevertheless, like PPCKs these *P. infestans *proteins are likely regulated at the transcriptional level.

### CK1 family

In other organisms this represents a typically small group of essential kinases that regulate repair, morphogenesis, and differentiation and are named after their ability to phosphorylate casein. *P. infestans *encodes three such proteins (Additional File 1 Table S1), which is similar to the number in most lower eukaryotes. As in other organisms, these kinases are unusual in that they lack the APE motif of subdomain VIII that is seen in other ePKs (Additional File 2 Fig. S1). Like other CK1 proteins, the *P. infestans *members have no additional domains although one (PITG_15970) contains extensive arginine, histidine, and serine-rich tracts in its C-terminal half. Similar regions are predicted in the *P. ramorum *ortholog of PITG_15970 and are present albeit to a lesser extent in the *P. sojae *ortholog.

### CMGC family

This group is named for cyclin-dependent kinase (CDK), mitogen-activated kinase (MAPK), glycogen synthase kinase (GSK) and Cdc2-like kinase (CLK), and also includes related kinases. *P. infestans *is predicted to encode 46 CMGC proteins (Additional File 1 Table S1). These include 17 CDKs, five DYRKs, 15 MAPK, and a small number related to each of the GSK, SRPK, RCK, CK2, and CLK subfamilies. The CMGC family represents 13% of all ePKs, and a similar fraction was found in other stramenopiles (Table 1).

CDKs were first identified as regulators of the cell cycle, with their activity being modulated by cyclins [33]. All sequenced eukaryotes express at least one CDK that contains the cyclin-binding PSTAIRE motif in subdomain III of the catalytic domain; when this binds cyclin, a conformational change is induced which enhances the ability of the kinase to bind ATP. Six of the 15 *P. infestans *CDKs (PITG_02446, PITG_06708, PITG_17990, PITG_18073, PITG_19450, and PITG_21617) contain PSTAIRE-like sequences and are defined as members of the CDK2 class (CdkA using plant nomenclature) which bind A- and B-type cyclins and play key roles at G1/S and G2/M transitions. A seventh kinase, PITG_20584, contains the PITSLRE motif instead of PSTAIRE which places it in a class associated with non-cell cycle roles such as RNA splicing [33].

For maximum activity CDKs must be phosphorylated in their activation loop, which spans subdomains VII and VIII of the catalytic domain. All 15 CDKs of *P. infestans *contain the expected serine or threonine substrates for phosphorylation at that location. Phosphorylation of the loop is performed by a CDK subfamily called CDK-activating kinases (CAK), which are also named CDK7 after the mammalian CAK. CDK7 also phosphorylates the carboxy-terminal domain of the large subunit of RNA polymerase II, and is a component of the general transcription factor TFIIH. Two *P. infestans *proteins match CDK7, PITG_07995 and PITG_21504. Another mechanism that regulates CDKs in other taxa involves the cyclin-dependent kinase regulatory subunit CKS, and *P. infestans *encodes a single CKS as PITG_12863.

Four *P. infestans *CDKs have the remarkable feature of bearing C-terminal regions which match strongly the Pfam domains for cyclins (Cyclin_N, Cyclin_C; Figure 2). As the combination of kinase and cyclin domains has not been described in any species, their structure was confirmed by RT-PCR to exclude the possibility that the gene-calling program had erroneously fused two adjacent genes. One of these four proteins, PITG_21617, also contains the cyclin-interacting PSTAIRE helix which might suggest it is autoactivated. However, all four may be enzymatically inactive due to the substitution of a catalytic aspartate in subdomain VIb with asparagine. A database search identified related genes in *P. ramorum, P. sojae*, *H. arabidopsidis*, the ciliates *P. aurelia *and *Tetrahymena thermophila*, and the apicomplexan *Theileria annulata*. These were not found in two other apicomplexans (*P. falciparum, T. gondii*) or the diatoms *T. pseudonana *and *P. tricornatum*. Interestingly, each gene contains the same aspartate to asparagine substitution in subdomain VIb, which suggests an important function, and perhaps the cyclin domain serves to regulate the stability of this innovative protein.

Another unusual CDK-like gene is PITG_14137. This also contains substitutions in the catalytic region that render it inactive, and cause it to cluster outside the CMGC group (Figure 1). The protein appears to be a fusion between an inactive kinase and an N-terminal histone-lysine N-methyltransferase. Orthologs with this usual structure exist in *P. ramorum *and *P. sojae*, but not other species including *H. arabidopsidis*.

A final comment related to CDKs is that *P. infestans *also encodes the expected cognate cyclins. These include six that may participate in cell cycle control such as three A-type, two B, and one H cyclins. Also detected were seven other cyclins that belong to the C, K, L, and M families, but these are not known to have cell cycle roles in other taxa [34]. For example, cyclin C binds the transcriptional regulator CDK8, which is known as CDKE in plants and represented in *P. infestans *as PITG_19235.

*P. infestans *has five proteins that cluster with the DYRK subfamily, which stands for dual specificity tyrosine-phosphorylated kinase. Three of these (PITG_00178, 14626, 08572) contain the tyrosine in the activation loop that is normally phosphorylated for full activity. DYRKs are notable since many catalyze both tyrosine- and serine/threonine-directed phosphorylation, besides being subjected to tyrosine phosphorylation themselves.

Another significant CMGC subfamily encodes mitogen activated kinases (MAPK). These typically link extracellular signals to cellular systems that control growth, development, and stress responses. MAPK proteins are activated by phosphorylation cascades that are often G-protein-stimulated and mediated by MAPK kinases (MAP2K), MAPKK kinases (MAP3K), and MAPKKK kinases (MAP4K). MAPK proteins in animals form three major groups named extracellular receptor kinases (ERK), c-Jun N-terminal and stress-activated protein kinases (JNK/SAPK), and p38 kinases, with the JNK and p38 pathways appearing metazoan-specific [10,35].

*P. infestans *is predicted to express 15 MAPK-like proteins, which are all ERK kinases. By comparison, humans contain a total of six ERK, JUN/SAPK, and p38 MAPKs while *A. thaliana *and *O. sativa *have 20 and 15, respectively, which are exclusively ERKs [36]. The diatom *T. pseudonana *has only five MAPKs, which are ERKs. The absence of JNK/SAPK and p38 from stramenopiles is consistent with reports that these are not distributed widely. All 15 MAPK-like proteins from *P. infestans *have the threonine that is the predicted MAP2K target within their activation loop. However, the TxY motif seen in other eukaryotes is only observed in 12 of the *P. infestans *MAPK-like proteins (11 TEY and 1 TDY), being absent from PITG_00254, PITG_01203, and PITG_15298. PITG_01203 and PITG_15298 instead contain a TEH motif, which is a conservative deviation from the consensus. PITG_00254 by contrast includes a dramatic change to TKH. The latter is also present in orthologs in *P. sojae*, *P. ramorum, H. arabidopsidis, T. pseudonana*, and *P. tricornatum *but not in apicomplexans or ciliates, making it a stramenopile-specific feature. Its maintenance even in the small diatom MAPK subfamily suggests that it serves an important role.

Another novel feature of a *P. infestans *MAPK is the presence of a PAS domain, which is a signal sensing fold present in archaea, eubacteria and eukaryotes. This is found N-terminal to the kinase domain in PITG_01203, and also in its *P. ramorum, P. sojae*, and *H. arabidopsidis *orthologs (Figure 2). The presence of PAS on an ePK has not been reported previously. It is not on any diatom, alveolate, or ciliate MAPK. The only other proteins in *P. infestans *that contain PAS domains are its three histidine kinases.

### STE kinases

These take their name from sterile-phenotype kinases of yeast, and includes the STE7/MAP2K, STE11/MAP3K, and STE20/MAP4K subfamilies which constitute much of the signaling cascade for MAPK activation. *P. infestans *has 15 STE kinases which include six MAP2K, five MAP3K, and four MAP4K proteins (Additional File 1 Table S1). This number is similar to that of yeast, but less than humans and plants (Table 1). Other stramenopiles including *H. arabidopsidis *and *T. pseudonana *have even fewer predicted STE kinases (five or six), and these are absent from the apicomplexans *P. falciparum *and *T. gondii *[37].

When considering how these STE kinases act in MAPK signaling, it is prudent to note that non-STE kinases may also participate in the cascade. In humans and *A. thaliana*, some members of the RAF subfamily of TKL kinases are MAP3Ks. However, no RAF kinases appear to exist in *P. infestans *where, as in yeast, MAP3K activity is likely limited to the STE11 subfamily. In addition, not all *P. infestans *STE kinases may be part of a MAPK cascade. Some organisms have two classes of STE11 proteins, namely the MAP3K class called MEKK and a non-MAP3K class called CDC [6]. While MEKK is ubiquitous, some species lack CDC. PITG_10045 belongs to the CDC class while the other STE11 proteins of *P. infestans *are MEKK-like MAP3Ks.

The ratio between MAPK, MAP2K, MAP3K, and MAP4K in *P. infestans *resembles that in *A. thaliana*, where there is near-stoichiometry between MAP2K, MAP3K, and MAP4K and a excess of MAPK. In contrast, in yeast each group has similar numbers while in humans MAPK and MAP4K are in excess. The implication of these ratios in *P. infestans *is that each MAP2K activates multiple MAPKs. That the numbers of MAP3K and MAP4K proteins are similar does not imply that the former are always activated by the latter, since other types of kinases can also activate MAP3Ks. Also, since the four predicted MAP4Ks have very different C-terminal regions, some may have functions unrelated to the MAPK cascade. This may resemble the case in plants, where many MAP4K-like proteins do not phosphorylate a MAP3K and instead act in other pathways [38].

One predicted MAP4K protein contains a C-terminal RhoGAP domain, which has not been found in any previous study of a non-oomycete. The domain is seen in both PITG_11253 and its orthologs from *P. ramorum *and *P. sojae*, but not in *H. arabidopsidis*. RhoGAP appears to be a protein interaction domain that is often associated with GTPase-activating protein complexes.

### TKL family

These kinases have sequence similarity to tyrosine kinases, but act biochemically as serine/threonine kinases. Such tyrosine kinase-like kinases (TKLs) exist in most eukaryotes except fungi [6,10]. *P. infestans *contains 139 TKL proteins, making it the largest family at 39% of ePKs (Additional File 1 Table S1). This is much more than other eukaryotes, except for plants (Table 1). While *P. infestans *contains proteins resembling members of TKL subfamilies seen in other species (IRAK, LISK, LRRK, RAF, RIPK, and STKR), most belong to oomycete-specific groups.

**Figure 3:**
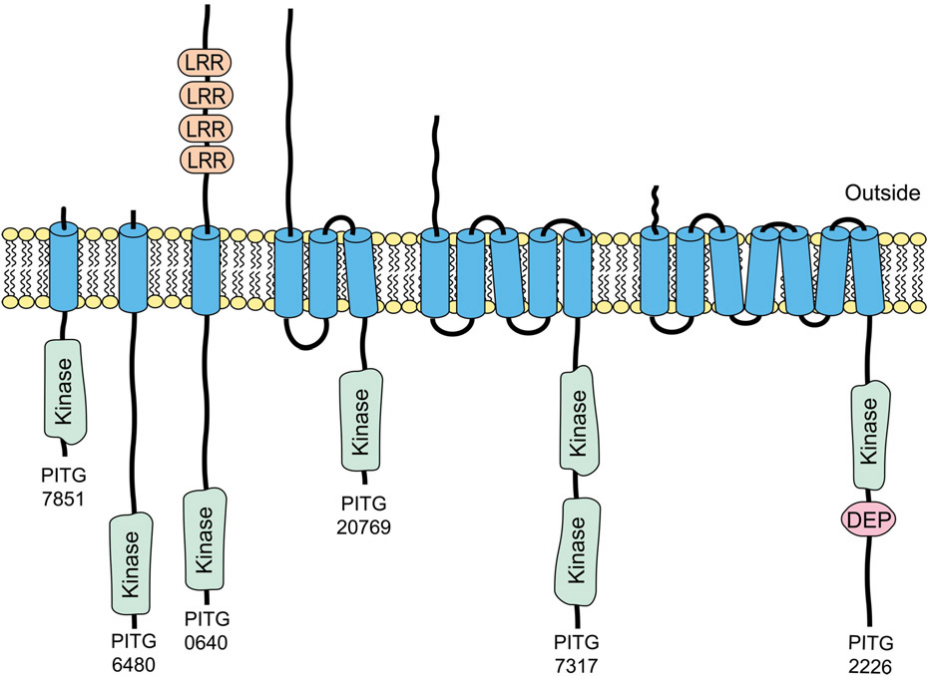
**Examples of transmembrane ePKs in *P. infestans***. Illustrated are six proteins displaying the diversity within the group, including proteins with single or multiple transmembrane domains, dual kinase domains, extracellular leucine-rich repeat (LRR) domains, or an intracellular DEP domain.

TKLs are the only *P. infestans *ePKs that contain transmembrane domains. Such proteins are marked by red circles in Figure 1 and the topologies of representative transmembrane TKLs are shown in Figure 3. These include proteins with very short predicted extracellular domains such as PITG_07851, proteins with long extracellular portions such as PITG_20769, and proteins with accessory domains in the extracellular or intracellular regions such as PITG_00640 and PITG_02226.

**Figure 4:**
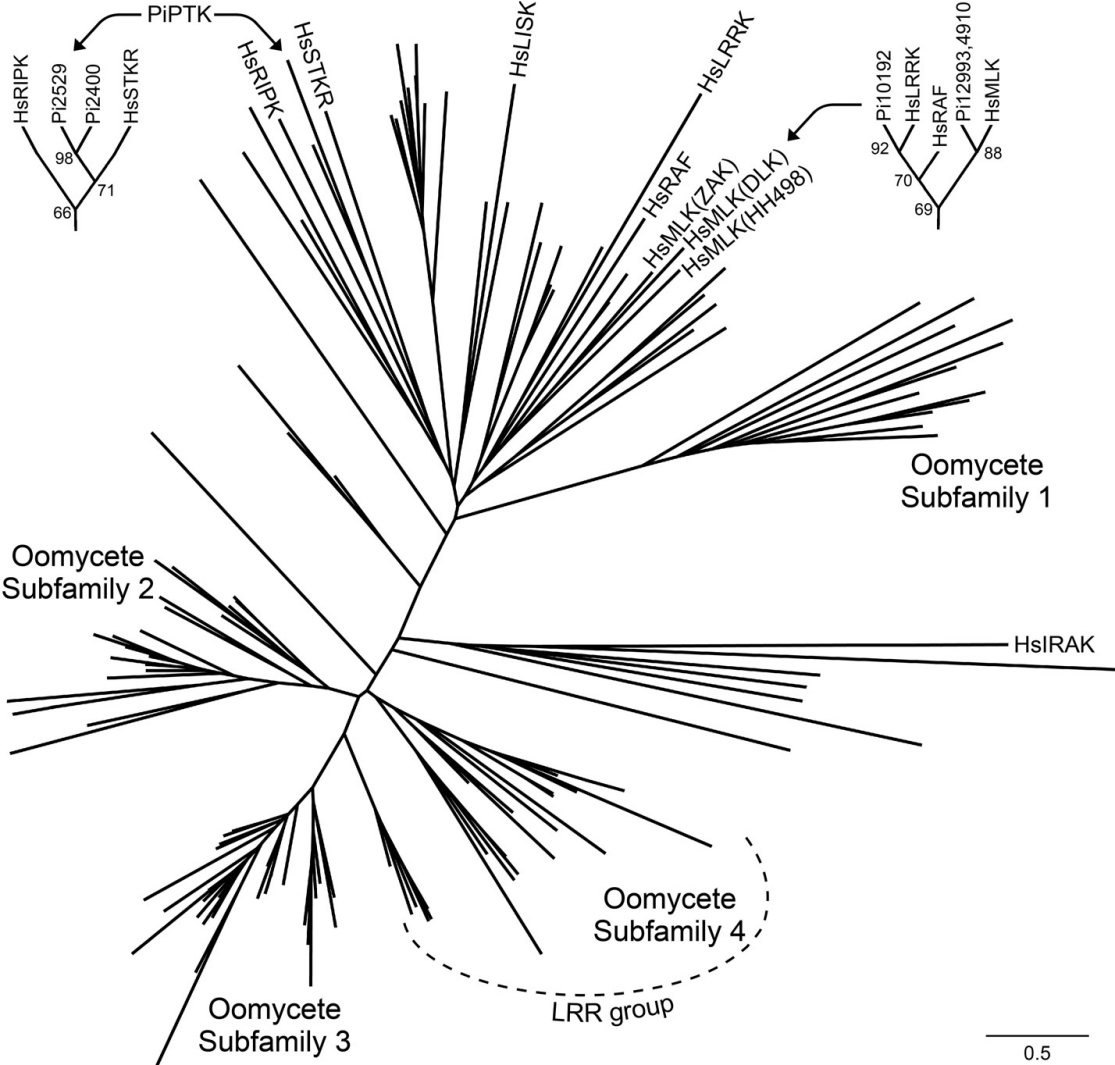
**Maximum likelihood tree of *P. infestans *TKL proteins and selected human TKLs**. Leaves with *P. infestans *proteins are unlabeled, while human proteins start with the prefix Hs. In the upper corners, subtrees are shown to help illustrate relationships between the *P. infestans *proteins and RAF kinases (upper right) or between the two putative TKs and human STKR and RIPK (upper left); numbers at nodes are aLRT confidence numbers from PhyML. Human gene names are based on the nomenclature at http://kinase.com. Subfamily assignments of the *P. infestans *proteins in the tree are in Additional File 1 Table S1.

Of the broadly distributed TKL subfamilies, the largest in *P. infestan*s is the mixed lineage kinase group (MLK) with eight members (Figure 4). In animals MLK proteins regulate apoptosis and stress signaling through p38/JNK; as the latter is absent from *P. infestans *these eight proteins presumably play different roles. In analyses based on either the catalytic region or whole protein, the eight MLK-like proteins of *P. infestans *only cluster loosely with human MLKs. The analysis does not indicate if the *P. infestans *proteins are closest to any particular eukaryotic TKL subfamily such as HH498, ZAK, or DLK, although none contain the leucine zippers and SAM domains of ZAK kinases or the dual leucine zippers of DLKs.

Possible members of other eukaryotic TKL subfamilies in *P. infestans *include 18 proteins that cluster near human LISK, LRRK, RIPK, or STRK sequences (Figure 4). However, most do not have the traditional structures of such proteins. For example, the LRRK-like kinase, PITG_10192, lacks the leucine-rich repeat domain that gives this group its name. Similarly, the two *P. infestans *proteins that cluster with LISK lack the N-terminal LIM domains typical of that group. The *P. infestans *proteins near metazoan STKR also lack the extracellular domains typical of such proteins, and most instead contain N-terminal transmembrane domains. Two proteins that cluster near human STKR (PITG_2400 and PITG_02529) are likely to function biochemically as tyrosine kinases, as will be discussed later.

*P. infestans *has five genes classified as IRAK kinases, which are named after interleukin-1 receptor-associated kinase (PITG_03773, PITG_03775, PITG_04568, PITG_7210, and PITG_22686). As shown in Figure 4 these form a coherent clade that is well-separated from other TKL subfamilies. In plants and animals, most are involved in the innate immune response where they are either membrane-spanning proteins that associate with pathogen recognition receptors (PRRs) or part of the receptors themselves. Of the five *P. infestans *IRAK proteins, all but PITG_22686 contain a transmembrane domain N-terminal to the kinase region. Curiously, all are predicted to be catalytically inactive. This partially resembles the case in humans, where two of the four IRAKs are predicted to be inactive yet still participate in the immune response [39]. Either these interact with signaling components independently of phosphorylation, or bioinformatic assessments of kinase activity are inaccurate. As *P. infestans *is not known to have an innate immune response, it will be interested to address the function of its IRAK proteins. Interestingly, no IRAK-like proteins are detected in *T. pseudonana*, *P. falciparum*, or *P. tetraurelia*.

The most striking feature of the TKL family in *P. infestans *is that 60% appear to be unique to oomycetes, and fall into four major clades marked as Oomycete Subfamilies in Figure 4. Relatives exist in *P. ramorum, P. sojae*, and *H. arabidopsidis *but not diatoms, apicomplexans, or ciliates. These presumptive oomycete-specific TKLs were also compared to nonmetazoan-specific TKL subfamilies from *D. discoideum *and plants, but no affinity was observed (not shown).

Most of the 13 members of Oomycete Subfamily 1 contain protein interaction domains. These may be used to regulate the kinase or contribute to signaling. For example, PITG_06288 contains 15 ankyrin repeats N-terminal to the kinase region, PITG_10645 contains 10 C-terminal Armadillo domains, PITG_11566 contains a C-terminal PDZ domain, and PITG_16204 contains eight C-terminal tetratricopeptide repeat (TPR) domains.

The remaining three oomycete-specific subfamilies appear similar to receptor kinases due to the presence of a signal peptide, transmembrane domains, and a cytoplasmic kinase domain. Twenty-two of the 25 proteins in Oomycete Subfamily 2 have one to six predicted transmembrane domains, with most also bearing signal peptides. Representatives shown in Figure 3 include PITG_06480, which lacks a sizable predicted extracellular domain, and PITG_20769 which contains a 364 amino acid extracellular domain. A similar situation exists in Oomycete Subfamily 3 where 27 of 35 proteins have membrane-spanning regions, and in Oomycete Subfamily 4 for 23 of 26 members. Notably, 16 of the transmembrane proteins in Oomycete Subfamily 4 group also contain extracellular leucine-rich repeats as illustrated for PITG_00640 in Figure 3.

It should be stressed that these putative receptor kinases are not related closely to animal receptor tyrosine kinases or plant receptor-like kinases, which form a well-supported monophyletic family along with the RAF group of TKL kinases [40]. In fact, no oomycete TKL is related to RAF as shown in the upper right portion of Figure 4. As part of a study of the diatom *T. pseudonana*, Montsant et al. also reported finding transmembrane leucine-rich repeat kinases in *P. ramorum *and *P. sojae*, as well as in *T.pseudonana *[20]. However, we find that the diatom proteins do not cluster with those oomycete-specific subfamilies. Bowler et al. also reported that the leucine-rich repeat kinases of the diatom *P. triconutum *are unrelated to proteins in *Phytophthora *[41]. It therefore appears that receptor-like kinases have appeared multiple times during evolution.

### Tyrosine kinases (TK)

The wide existence throughout eukaryotes of tyrosine phosphorylation has not been disputed, but whether this results from tyrosine kinases as opposed to dual-specificity kinases (such as DYLKs, which exist in *P. infestans*) has been controversial. TKs are a major component of the ePK superfamily in animals, and have been said to be a metazoan-specific invention [42]. Most studies report the absence of TKs from non-metazoans, but two papers suggested that primitive TKs exist in plants and green algae based on matches to a TK HMM or phylogenetic analysis [42,43].

**Figure 5:**
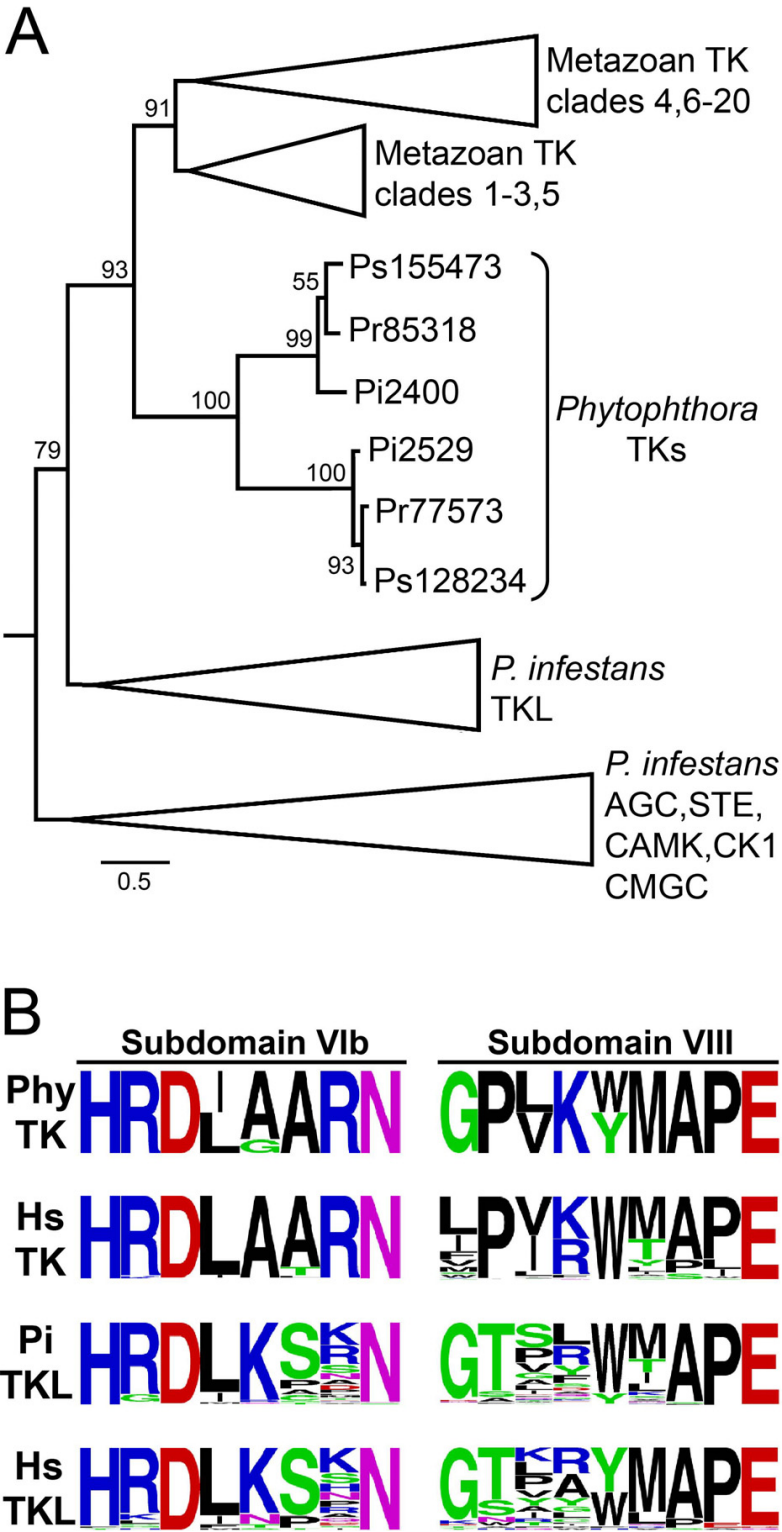
**Features of putative tyrosine kinases of *Phytophthora***. **A**, Maximum likelihood tree incorporating the TKs from *P. infestans *(Pi prefix) and their orthologs from *P. ramorum *(Pr) and *P. sojae *(Ps). Also included are three representatives of each of the *P. infestans *TKL, AGC, STE, CAMK, CK1, and CMGC groups, which are shown in collapsed branches to conserve space. The analysis also incorporated 60 metazoan TKs; to ensure that diverse TKs were analyzed, these included representatives of clades 1 to 20 as defined in a previous study [1]. Numbers at nodes are aLRT values from PhyML. **B**, Sequences of kinase subdomains VIb and VIII. Shown are relative amino acid frequencies for the predicted *Phytophthora *TKs (Phy; includes orthologs from *P. infestans, P. ramorum, P. sojae)*, along with human TKs. Also illustrated are the amino acid frequencies within those domains in *P. infestans *and human TKLs.

*P. infestans *proteins PITG_02400 and PITG_02529 resemble TKs, with the latter also containing transmembrane domains near its N-terminus. In our initial analyses these clustered with TKL kinases (Figure 1). When included in phylogenetic trees with *P. infestans *and human TKLs, these two proteins had affinity to human STKR albeit with only modest branch support (Figure 4 upper left corner). However, when the catalytic domains of PITG_02400 and PITG_02529 were searched against a database of metazoan kinases, the best hits were against TKs with *E*-values of 10^-23 ^and 10^-33^, respectively. When tested against the Kinomer HMMs [44], the two *P. infestans *proteins best-matched the TK model with *E *values of 10^-15 ^and 10^-45^, respectively. In phylogenies with metazoan TKs and the other *P. infestans *ePKs, PITG_02400 and PITG_02529 clustered with the TKs with strong branch support (Figure 5A). *P. ramorum *and *P. sojae *orthologs showed similar placement in the trees, while no orthologs were found in *H. arabidopsidis*.

Closer examination of the catalytic regions of PITG_02400 and PITG_02529 provided more evidence for these being tyrosine kinases. Prior studies indicated that subdomains VIB and VIII contribute to substrate recognition, with most serine/threonine kinases having HRD(I/L)KxxN in subdomain VIb and tyrosine kinase having HRD(I/L)AARN. As shown in Figure 5B, the two putative *P. infestans *TKs and orthologs from *P. ramorum *and *P. sojae *contain the latter. PITG_02400 and PITG_02529 also match the TK signature in subdomain VIII, having proline in the second position instead of threonine. These can be compared to the sequences of these subdomains in other human and *P. infestans *ePKs in more detail in Additional File 2 Fig. S1.

This data provides the strongest support yet for the existence of TKs outside metazoans. The putative *A. thaliana *TKs described previously [44] had *E *values against the TK HMM of only 10^-13 ^compared to 10^-45 ^for PITG_02529. We observe that those kinases lack the AARN at the end of subdomain VIb, and another genome-wide study in *A. thaliana *found that all of the plant kinases contained the KxxN motif associated with serine/threonine kinases [45]. Also, unlike the putative *A. thaliana *TKs which are predicted to be catalytically dead, the *P. infestans *TKs contain the lysine in subdomain II and aspartates in subdomains VIb and VII that are considered required for phosphotransfer activity. One previous study also suggested that *P. infestans *might contain a TK based on the clustering of an EST-derived sequence with TKs, albeit with weak branch support [42]. Our analysis of the full-length gene suggests it is a serine/threonine kinase, however.

It therefore appears that TKs predated the radiation of eukaryotic life forms, although convergent evolution can not be excluded. The extent of the importance of tyrosine phosphorylation in oomycetes remains to be elucidated. Only four proteins in *P. infestans *contain plausible SH2 phosphotyrosine-binding domains and none have PTB phosphotyrosine-binding domains. Dual-specificity phosphatases have been detected, however, which are an indicator of phosphotyrosine signaling [46].

### OTHER family

This comprises ePKs that do not fit well into the above-described groups, but which nonetheless are typically well-conserved in eukaryotes. *P. infestans *contains 35 OTHER kinases (Additional File 1 Table S1). These include ePKs involved in cell division such as four Aurora, six NEK, two POLO-like (PLK), and two WEE kinases. Also detected were four NAK kinases which in other species regulate the cytoskeleton, one VPS15 kinase which participates in protein sorting, a WNK kinase which regulates ion homeostasis, and five PEK/GCN2 kinases which control translation initiation and participate in the starvation response. Two ULK kinases were also detected, which in yeast control autophagy.

Some workers suggested that most of these kinases can be placed in the main families. We have not chosen that approach in this study since most group with each other in phylogenetic analysis (Figure 1). The exceptions are Aurora and POLO, which have affinity to the CAMK family.

### Expression pattern of ePKs

Microarray and qRT-PCR analysis was used to measure mRNA levels in hyphae, asexual sporangia, and swimming zoospores to obtain more insight into the function of the kinases. By mining data from our prior microarray study [47], reliable signals were obtained for 221 ePK genes in one or more of the three developmental stages. The remainder were either not represented or gave poor signals on those microarrays, and to obtain data from these qRT-PCR was performed. In total, expression could be measured reliably for 293 kinases in the three developmental stages by combining both approaches. Five genes were studied using both methods, which revealed similar patterns of expression.

**Figure 6:**
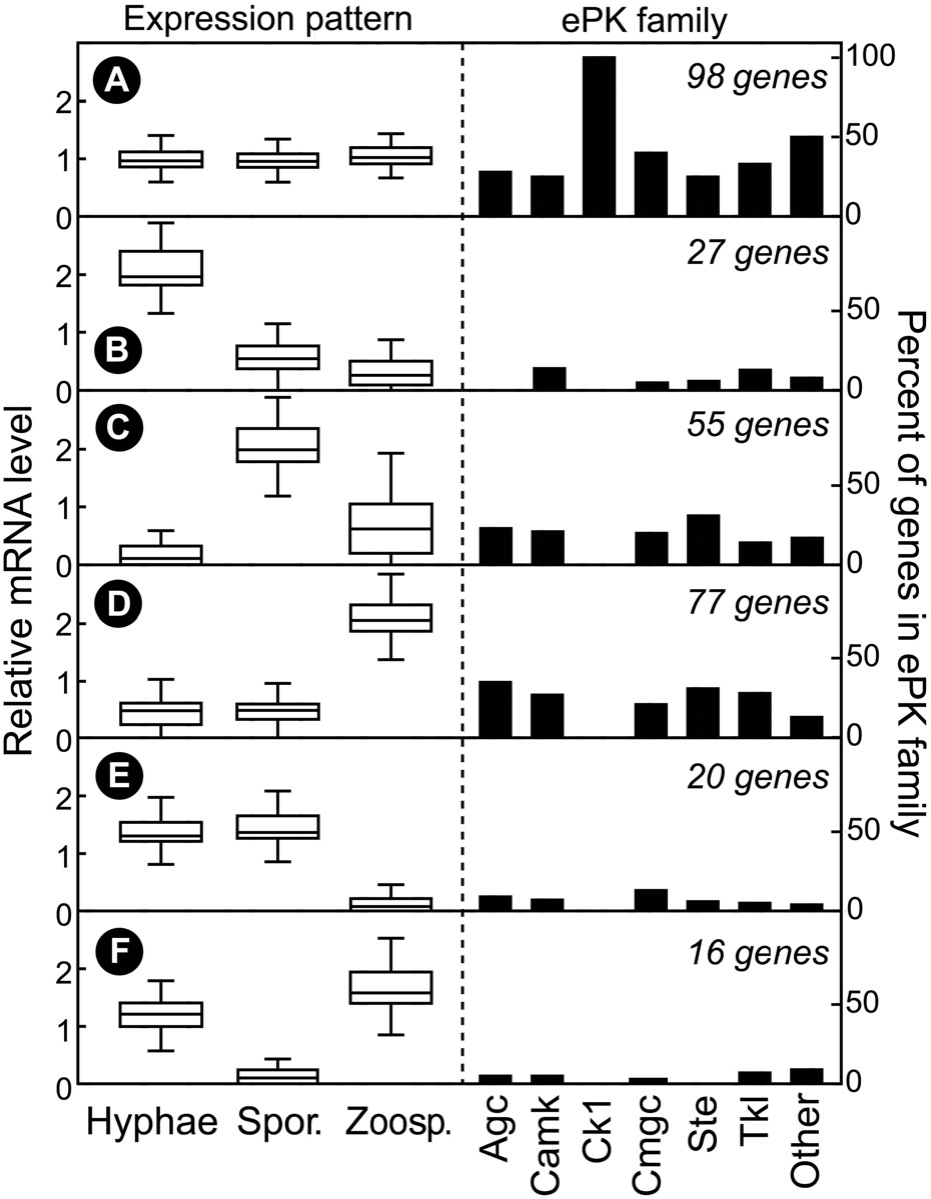
**Expression patterns of *P. infestans *ePKs**. Data from microarray and qRT-PCR experiments were pooled, per-gene normalized, and subjected to hierarchical and K-means clustering. Six main clusters (A-F) were identified based on mRNA levels in hyphae, sporangia, and zoospores. Shown on the left are box-plots of the expression levels in each tissue, with horizontal lines matching the 0, 25, 50, 75, and 100th percentiles of the genes in each group. Shown on the right are the percent of ePKs in each of the major families that reside in clusters A to F.

A compilation of the data is presented graphically in Figure 6 and in more detail in Additional File 1 Table S3. Levels of mRNA for 194 genes, or 66% of measured ePKs, exhibited >2-fold differences between hyphae, sporangia, or zoospores at *P *< 0.05, based on two biological replicates. Six main patterns were detected. Ninety-eight genes showed little change between stages (Figure 6A), 27 were mostly hyphal-specific (Figure 6B), 55 were upregulated in sporangia (Figure 6C), and 77 were induced in zoospores (Figure 6D). Smaller numbers of genes were down-regulated in zoospores or sporangia (Figure 6E, F).

While many ePKs are subjected to strong transcriptional regulation during development, not all families show the same patterns. This is illustrated on the right side of Figure 6 which shows the proportion of each expression pattern within each family. For example, the CK1 family shows little change between stages, AGC kinases lack hyphal-specific members, and most STE kinases are induced in sporangia or zoospores. The latter observation implies that some MAPK pathway branches may be important in spore development or germination.

Another observation that may shed light on how life-stages are controlled in *P. infestans *involves two genes encoding WEE kinases, which in other species block entry into mitosis. PITG_00477 mRNA is upregulated 10-fold in sporangia and PITG_10062 is zoospore-induced, which may explain why mitosis is dormant in these spores. Also highly upregulated in zoospores is PITG_18073, which is a cyclin-dependent kinase. It is interesting to speculate whether this gene regulates nuclear or cellular dynamics in zoospores or germinating zoospore cysts. One more story of note involves the sporulation-specific kinase PITG_13567. This encodes a NEK kinase, which in other species regulates the formation and stability of the axonemal microtubules of flagella [48]. Perhaps this protein also plays such a role in sporangia, which already contain all proteins needed for zoosporogenesis [49]. Other kinases with intriguing potential functions can be extracted from the data but are too numerous to describe here.

Besides suggesting how kinases regulate development, the expression data can help indicate whether the ePK gene models actually lead to a protein; in any genome project, not all predicted proteins are necessarily expressed. Based on data from the microarray and qRT-PCR studies, and searches of databases of *P. infestans *expressed sequence tags for matches to the gene models, it appears that at least 327 of the 354 *P. infestan*s ePK genes are transcribed. Evidence of expression was also obtained for 27 of the 32 predicted catalytically inactive kinases.

### Genomic organization of ePKs

*P. infestans *has a large and complex genome, in which the total size is 240 Mb and 70% of sequences are repetitive [18]. Many gene families appear to have expanded through mechanisms such as unequal crossing over. This includes ePKs where 66 are organized in 23 clusters of 2 to 13 members; a cluster is defined as two or more genes of similar sequence that reside within two genes of each other on a genomic scaffold.

**Figure 7:**
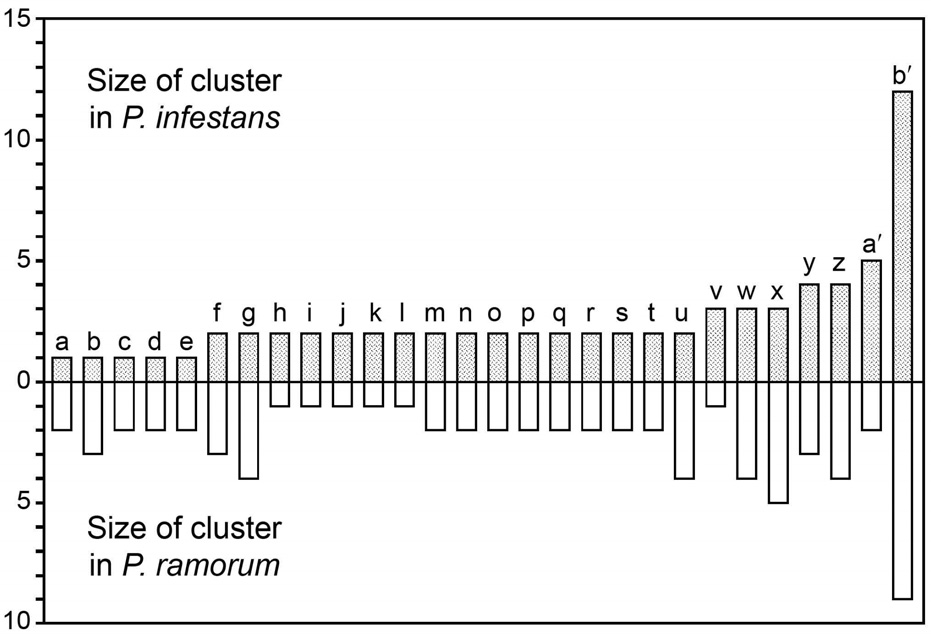
**Clusters of ePKs in the genomes of *P. infestans *and *P. ramorum***. A survey of the distributions of ePKs along supercontigs revealed 28 clusters in one or both species (a to z, a' to b'). Shown are the number of genes within orthologous clusters.

A comparison of *P. infestans *and *P. ramorum *indicate that most ePK clusters predate speciation. Of ePKs within the 23 clusters of *P. infestans*, orthologs of at least 17 also comprise clusters in *P. ramorum *(Figure 7). The opposite analysis reveals that 22 clusters exist within *P. ramorum*, with 17 conserved in *P. infestans*. These are approximate values since both species have draft genomes with some short contigs.

While the number of clusters in each species are similar, many show dynamic changes. For example, cluster a' contains five ePKs in *P. infestans *but only two in *P. ramorum*, while cluster g has four members in *P. ramorum *and two in *P. infestans*.

These differences may be important for biological diversification. Remarkably, these expansions and reductions are balanced such that kinome sizes are nearly identical between *P. infestans *(354) and *P. ramorum *(352). Orthologous pairs can also be identified for nearly every kinase in these species, if one accounts for family expansion. Their kinomes are also more similar in size than their proteomes, as *P. infestans *is predicted to have 17,797 genes and *P. ramorum *14,451. The most common clustered ePK gene encodes a TKL kinase. This may explain why this family is the largest and why it contains the most catalytically inactive members, since mutations may be tolerated more in a gene copy after duplication. This does not necessarily mean that most catalytically inactive TKL proteins lack a function. Most also contain transmembrane domains and therefore resemble the receptor tyrosine kinases of metazoans, where some pseudokinases act as regulatory partners. For example, in humans the HER2 kinase assumes its active conformation only after associating with the HER3 pseudokinase [21].

### Comparison of P. infestans and H. arabidopsis kinomes

The *P. infestans *and *H. arabidopsidis *kinomes were comprehensively compared to more thoroughly study how kinomes evolved over a broader evolutionary distance. *H. arabidopsidis *is in a different family of the Class Oomycota (Peronosporaceae versus Pythiaceae), although not very distant compared to other oomycetes [50]. *H. arabidiopsidis *is distinguished from many oomycetes including *P. infestans *in that the former has lost the ability to produce zoospores and can not grow on artificial media which may indicate a loss of metabolic or regulatory pathways [51]. The current genome release of *H. arabidopsidis*, based on 9.5-fold Sanger coverage, measures its gene content at about 90% of *P. ramorum *and 75% of *P. infestans*. Much of the variation in gene number has been attributed to differences in families of effector proteins that modulate interactions with plants [52]. Gene content does not correlate to genome size, as the downy mildew genome is larger than that of *P. ramorum *(77 versus 65 Mb).

The *H. arabidopsidis *ePK kinome comprises 207 proteins, which is 42% less than *P. infestans *and exceeds the 25% difference in their total genes. Apparently, ePK genes were lost from the downy mildew or families expanded in *P. infestans*. The possibility that the small appearance of the *H. arabidopsidis *kinome is an artifact of poor gene calling or assembly was considered but excluded. In multiple cases where an ortholog was absent, we searched unsuccessfully for "missing" genes in noncoding regions of the assembly and unplaced reads. Also, as will be noted below, the absent kinases are preferentially from certain subfamilies. It is possible that the challenge of assembling diverged alleles in diploids led to an overestimation of the *P. infestans *kinome, but only four of its ePKs were on small contigs or contig edges which might suggest this.

Additional insight into the evolution of the two kinomes was revealed by comparing the number of ePKs per family (Table 1) and subfamily (Table 3). The *P. infestans *ePKs that contain orthologs in the downy mildew are also marked with blue circles in Figure 1. These data reveal that the loss of kinases in the downy mildew is uneven. Only the CK1 family has the same size in both species, with the rest being 20 to 60% reduced in the downy mildew. Some differences are remarkable and have significant biological implications. For example, *P. infestans *is predicted to encode five STE11 MAP3Ks compared to only one for the downy mildew.

Notable differences that might be related to the absence of zoospores from the downy mildew are seen in the NAK and NEK families. Four and one NAKs are detected in *P. infestans *and *H. arabidopsidis*, respectively. NAKs regulate cytoskeleton dynamics [53], which is important during zoosporogenesis and zoospore cyst germination. Moreover, two of the NAKs that are missing from the downy mildew are transcriptionally induced in *P. infestans *sporangia, which is the stage that forms zoospores. Regarding the NEK subfamily, *P. infestans *and *H. arabidopsidis *are predicted to encode six and three such proteins, respectively. NEKs are known to regulate flagella [48], and at least one gene missing from the downy mildew is upregulated in *P. infestans *sporangia. However, not all genes missing from the downy mildew exhibited spore-specific expression in *P. infestans*.

While many subfamilies exhibit large differences between the species, others were present in equal numbers. For example, this was the case with POLO and WEE. The TKL subfamily IRAK also had identical numbers. In contrast, other TKL subfamilies showed dramatic changes. For example, the OS1 subfamily is reduced from 13 to 2 members in the downy mildew compared to *P. infestans*, and OS3 is trimmed from 33 to 7. Since TLKs are the most likely ePKs to reside in clusters, their expansion in *P. infestans *through unequal mitotic crossing over might explain the differences.

### Atypical protein kinases

Many species express so-called atypical protein kinases (aPKs), which phosphorylate proteins but are not members of the ePK group [2,3]. Some have weak similarity to ePKs, while others have unique evolutionary histories and catalytic mechanisms. Humans encode a total of 20 aPKs while yeast makes nine. *P. infestans, P. ramorum*, and *H. arabidopsidis *encode 18, 20, and 18 aPKs, respectively (Additional File 1 Table S2).

RIO kinases have some similarity to the ePK catalytic domain, but interact differently with ATP and lack the standard peptide binding region. RIO is found in organisms ranging from archaea to eukaryotes, and participates in ribosome biogenesis and some cell cycle events. *P. infestans *encodes four RIO proteins, which can be classified as three RIO1 and one RIO2 based on diagnostic features in their N-termini [2]. *P. ramorum *also has four RIO kinases, while *H. arabiodopsidis *has three. An explanation for the "missing" gene in the downy mildew is that one gene duplicated early in the *Phytophthora *lineage, since two similar RIO genes reside near each other in *P. infestans*, *P. ramorum*, and *P. sojae*. Retroelement-like sequences comprise much of the intervening DNA which hints at the process underlying the duplication.

Alpha kinases are named based on their habit of phosphorylating alpha helices, and are known to regulate transcription elongation and ion channels. They have minor sequence similarity but stronger structural similarity to the ePK catalytic fold. A check of oomycetes and neighboring taxa reveals a checkered pattern of distribution of these proteins. *P. infestans, P. ramorum*, and *H. arabidopsidis *each encode three Alpha kinases, which are unlinked in their genomes. The ortholog group exemplified by PITG_06533 contains a weak match (*E *= 10^-4^) to a N-terminal von Willebrand factor type A (VWA) domain, which may be a metal ion-dependent protein adhesion site. Alpha kinases with the VWA domain have been described previously in humans and fungi. We found Alpha kinases in the moss *Physcomitrella patens*, albeit without the VWA domain, but not in higher plants. Diatoms also contain Alpha kinases without VWA domains and the ciliate *P. tetraurelia *encodes several Alpha kinases including some with VWA. None were found in the apicomplexans *T. gondii *or *P. falciparum*.

Phosphoinositide 3' kinase-related kinases (PIKK) have some structural similarity to ePKs, but utilize a divergent catalytic mechanism and are more related to lipid kinases. PIKKs only phosphorylate proteins and are distinguished from lipid kinases by the presence of N-terminal FAT and C-terminal FATC domains. These proteins are widely distributed in eukaryotes, and help coordinate cellular responses to stress. *P. infestans*, *P. ramorum*, and *H. arabidopsidis *each encode four PIKKs, which are unlinked to each other in the genome. Two in each species contain a rapamycin binding domain, which defines them as relatives of the mammalian mTOR protein. In addition, the species contain two proteins that appear PIKK-like, but lack either the FAT (PITG_05423) or FATC domains (PITG_03571).

Pyruvate dehydrogenase kinase (PDHK) is a mitochondrial protein which regulates the activity of pyruvate dehydrogenase. Unlike the kinases described previously, it phosphorylates histidine. PDHKs can be identified by the presence of histidine kinase-ATPase and mitochondrial branched-chain alpha-ketoacid dehydrogenase kinase domains. *P. infestans, P. ramorum *and *H. arabidopsidis *each encode four PDHKs. While two of the PDHKs are adjacent to each other in *P. ramorum *and *H. arabiodopsidis*, this is not the case in *P. infestans *which suggests a post-speciation genome rearrangement.

Histidine kinases (HK) are believed to have evolved in bacteria and spread by horizontal transfer to eukaryotes [3]. They are found in most eukaryotes except animals. Bacterial HK systems are generally composed of an autophosphorylating histidine kinase and a separate response regulator protein, but these are usually on the same molecule in eukaryotes. The size of the HK family exhibits some flexibility in oomycetes as three, five, and four were found in *P. infestans, P. ramorum*, and *H. arabidopsidis*, respectively. In each species the genes reside in one or two clusters, which suggests a mechanism underlying the different numbers. Each protein contains both the kinase and response regulator domains typical of eukaryotic HKs, but not the transmembrane domain seen in some HKs. Each oomycete HK contains a tandem array of N-terminal PAS folds, which is a cofactor sensor domain. The three *P. infestans *proteins contain 10, 15, and 21 PAS domains, *P. ramorum *proteins have up to 20, and *H. arabidopsidis *proteins have up to 11. The size of these arrays are exceptional since eukaryotic HKs with PAS folds typically contain one to three, and the same is true for most bacterial HKs although up to 11 are reported in *Geobacter*.

Several data raised the possibility that distinct horizontal transfer events provided HK to oomycete and non-oomycete lineages. For example, the size of the oomycete PAS arrays resemble those of bacteria. Also, the HK of the diatom *T. pseudonana *has only one PAS domain, plus a GAF (phytochrome) domain that is also in plant HKs but not oomycetes. PAS domains are also absent from HKs of the ciliates *P. tetraurelia *and *T. thermophila*. Finally, the apicomplexans *P. faliciparum *and *T. gondii *lack HKs, although *Cryptosporidium parvum *may contain one HK-like protein although response regulator and PAS domains are absent. Phylogenetic analyses using whole protein sequences, or separate analyses of the histidine kinase phosphoacceptor, histidine kinase ATPase, and response regulator domains did not provide a clear resolution about whether oomycetes acquired HKs independently, however.

While aPKs are not the central focus of this paper, they provide a useful control for comparing the kinomes of *Phytophthora *and *H. arabidopsidis*. While major changes in ePK numbers were observed, aPKs were nearly identical. The small differences in aPKs appear to be attributable to repeat expansion, which apparently also influenced the evolution of the ePK families.

## Conclusions

*P. infestans *contains a large kinome compared to that of most other lower eukaryotes, including fungal plant pathogens that occupy similar environmental niches but typically express only about 100 ePKs and less than 10 aPKs [54]. The comparison of *Phytophthora *and *Hyaloperonospora *also revealed diversity within oomycetes, which may underlie their biological differences. It was notable that TKs were detected only in *Phytophthora*, which in itself is a striking discovery since there are few examples of TKs outside metazoans. This underscores the value of including diverse eukaryotes in studies of kinase evolution besides the animal, fungal, and plant models. Features shared between oomycete and plant ePKs (and often alveolate ePKs as well) such as the CDPK-like and calcineurin-regulated SnRK3 subfamilies also help to solidify theories concerning the transfer of genes from a common ancestor or endosymbiont.

Functional studies have been performed on only one oomycete kinase [55], so this paper has minimized speculation about their cellular roles. Nevertheless the data hint about which may be worth examining to learn more about novel aspects of oomycete biology. For example, studies of spore-specific kinases in subfamilies with roles in cytoskeleton dynamics might help illuminate zoosporogenesis, and examination of the receptor-like kinases might reveal signaling mechanisms at the plant-pathogen interface. These kinases and their associated signaling pathways might also be useful targets for crop protection chemicals, or drugs against maladies caused by the animal-pathogenic oomycetes. Kinases are subjects of many drug discovery activities in medicine [56].

## Methods

### Discovery of protein kinase genes and gene model correction

*P. infestans *genomic sequences (assembly version 2) and gene models were obtained from the Broad Institute of MIT and Harvard http://www.broadinstitute.org/science/data. ePK candidates were identified by searching these with HMM profiles of kinase domains, using TBLASTN to search the assemblies using catalytic domains from selected kinases (from *Saccharomyces cerevisiae, Arabidopsis thaliana*, humans, and oomycetes), and checking for keywords within lists of BLAST hits against GenBank. This resulted in a list of about 370 ePK candidates. Several ePKs appeared to be duplicates resulting from incorrect assembly and were discarded; these typically involved cases where the two genes had identical sequences, with one based on a large contig and the other on a very short contig. ePK pseudogenes were predicted based on the presence of internal stop codons, frameshift mutations within the catalytic domain, or deletions in otherwise well-conserved regions. aPKs were found using similar approaches.

Gene models were assessed by using expressed sequence tag data, checking for questionable features, and making comparisons to *P. ramorum *and *P. sojae *gene models (v. 2.0) available at http://vmd.vbi.vt.edu/toolkit/. Reverse transcription-polymerase chain reaction (RT-PCR) was also used to test models containing novel domain combinations which might have resulted from the artificial fusion of two genes. In total, more plausible gene models were created for 60% of *P. infestans *genes.

Kinases were also obtained from the databases of *H. arabidopsidis *(v. 8.3), *P. ramorum*, and *P. sojae*. This entailed keyword searches and TBLASTN searches of gene models, assemblies, and unassembled reads. Due to the potential of errors in gene models, a low-stringency threshold *E *value of 10^-10 ^for matches against the Pfam HMM for ePKs was used to identify the kinases. Data from *T. pseudodonana *were obtained from the Joint Genome Institute (v. 3; http://genome.jgi-psf.org/) and classified. While a prior report identified 190 gene models tagged with the IPR000719 annotation for ePKs [20], about 20% had very weak *E *values and stronger matches in GenBank against non-ePKs, and were consequently eliminated from our analysis. The proteomes of other organisms were not analysed systematically, but were searched at GenBank, EuPATHdb http://eupathdb.org, or the Joint Genome Institute. Orthologs were identified using a combination of reciprocal best BLAST and phylogenetics methods.

### Classification of kinases

Kinases were categorized according to the taxonomy established by Hanks and Hunter [1] using three methods in parallel. One involved comparing the sequences against a database of metazoan kinases that had been classified into families and subfamilies http://kinase.com. A second matched the unknowns to HMM models for different kinase families using Kinomer http://www.compbio.dundee.ac.uk/kinomer. When contradictions occurred, a final determination was made by phylogenetic analysis against representative kinases.

### Protein domain analysis

Proteins were searched for matches against the Pfam and SMART databases http://pfam.sanger.ac.uk, http://smart.embl-heidelberg.de using an *E *threshold of 10^-5^. *P. infestans *gene models having weak matches to the protein kinase domain (*E *> 10^-10^) were rechecked to help eliminate errors. Transmembrane domains were identified using the TMHMM server at http://www.cbs.dtu.dk/services/TMHMM/, and signal peptides using PSORT at http://psort.ims.u-tokyo.ac.jp/form.html.

### Phylogenetic analysis

Alignments of protein sequences were performed using the SEAVIEW implementation of MUSCLE using default parameters [57]. Maximum likelihood trees were made from these alignments using PhyML, using the LG substitution model and collecting SH-like aLRT data for branch support. These were compared with neighbor-joining trees constructed using BioNJ using 100 bootstrap replicates for alignments of the total kinome, or 500 replicates for individual groups of kinases. Trees were visualized using the FigTree program.

### Kinase expression

mRNA levels during development were calculated from Affymetrix microarray data [47], which are deposited in NCBI GEO as series GSE9623, or data generated for this study by qRT-PCR. This employed DNAse-treated RNA from nonsporulating vegetative hyphae grown on rye-sucrose broth, freshly harvested and unchilled sporangia from 7-day cultures, and swimming zoospores released from the sporangia prepared as described [47]. At least two biological replicates of each tissue were used. Hot-start *Taq *polymerase (Applied Biosystems, Foster City, California USA) was used in amplifications with primers targeted to the 3' portions of genes (150-225 nt amplicons; Additional File 1 Table S4), with the intercalation of SYBR Green as a reporter. cDNA levels were normalized based on primers for a constitutively expressed gene encoding ribosomal protein S3a. Expression was determined by the ΔΔCT method from triplicate reactions. Relative expression data from microarrays and qRT-PCR were pooled and analyzed using GeneSpring (Agilent Technologies, Foster City, California USA).

### Calcium binding assays

Recombinant PITG_08008 was prepared using pMAL-C2x as a fusion with maltose binding protein. The recombinant protein and maltose binding protein alone were purified on amylose columns and tested for binding to ^45^Ca^2+ ^using an overlay assay [58].

## List of abbreviations

aPK: atypical protein kinase; ePK: eukaryotic protein kinase; HMM: hidden Markov model; qRT-PCR: quantitative reverse transcription-polymerase chain reaction.

## Authors' contributions

HSJ carried out the global analyses of protein kinases and performed expression studies. AAF analysed subsets of kinases and other proteins, tested gene models, and performed biochemical studies of kinases. All authors participated in writing the manuscript and approved the manuscript.

## Supplementary Material

Additional File 1 **Table S1**. Sequence, structure, and classification of ePKs from P. infestans. Table S2. Sequence, structure, and classification of aPKs from P. infestans. Table S3. Expression data for ePKs from P. infestans. Table S4. Primers used for qRT-PCR. Click here for file

Additional File 2 **Fig**. **S1.** Amino acid composition of catalytic region subdomains VIb and VIII in the seven main groups of kinases from P. infestans and humans. Click here for file
