# Supplementary material for: The kinome of Phytophthora infestans reveals oomycete-specific innovations and links to other taxonomic groups
Source: BMC Genomics. 2010 Dec 9;11:700. doi: 10.1186/1471-2164-11-700 (PMC3019232; doi:10.1186/1471-2164-11-700)
Supplement: Additional File 2 — Fig. S1. Amino acid composition of catalytic region subdomains VIb and VIII in the seven main groups of kinases from P. infestans and humans. [file 1471-2164-11-700-S2.PDF]

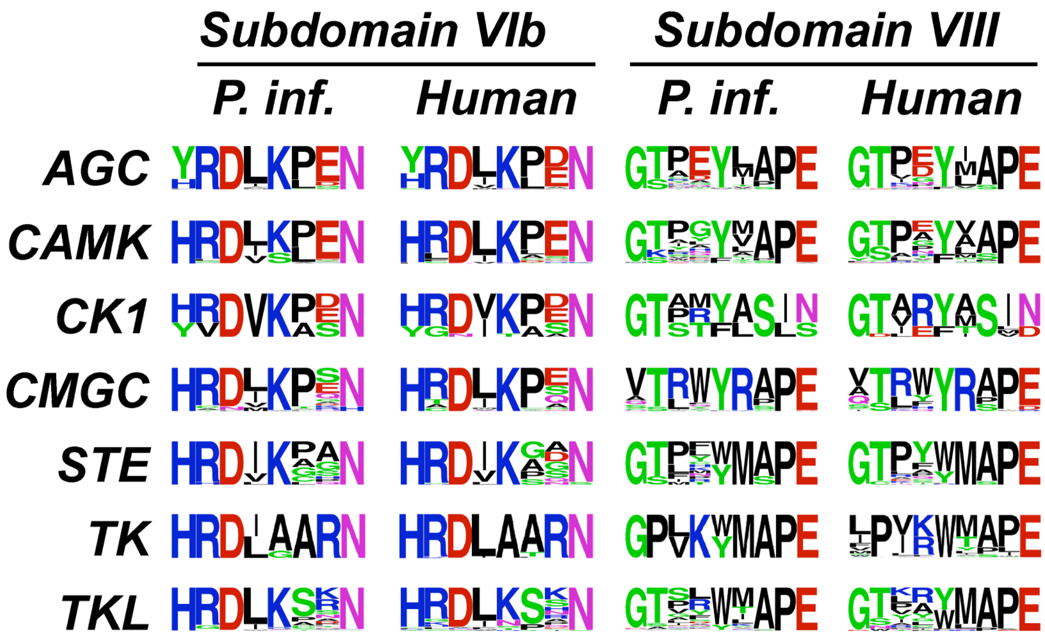

Figure S1. Amino acid composition of catalytic region subdomains VIb and VIII. Shown are frequency plots of amino acids within the two subdomains from the indicated ePK families. Data are from the human and *P. infestans* kinomes, except for the TK group; since only two TKs were detected in *P. infestans,* the data includes orthologs from *P. ramorum* and *P. sojae* in order to provide more comprehensive information. Human ePK data was extracted from the alignments archived at kinase.com.
